# Enteric glial cell diversification is influenced by spatiotemporal factors and source of neural progenitors in mice

**DOI:** 10.3389/fnins.2024.1392703

**Published:** 2024-08-29

**Authors:** Marie A. Lefèvre, Zoé Godefroid, Rodolphe Soret, Nicolas Pilon

**Affiliations:** ^1^Molecular Genetics of Development Laboratory, Département des Sciences Biologiques, Université du Québec à Montréal (UQAM), Montréal, QC, Canada; ^2^Centre D’excellence en Recherche sur les Maladies Orphelines-Fondation Courtois (CERMO-FC), Université du Québec à Montréal, Montréal, QC, Canada; ^3^Département de Pédiatrie, Université de Montréal, Montréal, QC, Canada

**Keywords:** enteric glial cells, enteric nervous system, myenteric plexus, submucosal plexus, schwann cell precursors, postnatal development, topo-morphological subtypes, cell lineage tracing

## Abstract

Previously focused primarily on enteric neurons, studies of the enteric nervous system (ENS) in both health and disease are now broadening to recognize the equally significant role played by enteric glial cells (EGCs). Commensurate to the vast array of gastrointestinal functions they influence, EGCs exhibit considerable diversity in terms of location, morphology, molecular profiles, and functional attributes. However, the mechanisms underlying this diversification of EGCs remain largely unexplored. To begin unraveling the mechanistic complexities of EGC diversity, the current study aimed to examine its spatiotemporal aspects in greater detail, and to assess whether the various sources of enteric neural progenitors contribute differentially to this diversity. Based on established topo-morphological criteria for categorizing EGCs into four main subtypes, our detailed immunofluorescence analyses first revealed that these subtypes emerge sequentially during early postnatal development, in a coordinated manner with the structural changes that occur in the ENS. When combined with genetic cell lineage tracing experiments, our analyses then uncovered a strongly biased contribution by Schwann cell-derived enteric neural progenitors to particular topo-morphological subtypes of EGCs. Taken together, these findings provide a robust foundation for further investigations into the molecular and cellular mechanisms governing EGC diversity.

## 1 Introduction

The enteric nervous system (ENS) is an integrated neural network that extends throughout the gastrointestinal tract. In mammals, the ENS is mainly subdivided into the myenteric and submucosal plexuses, each consisting of interconnected ganglia containing both enteric neurons and enteric glial cells (EGCs). Each plexus serves overlapping yet distinct functions (Furness, 2012; Schneider et al., 2019; Sharkey and Mawe, 2023). For instance, the myenteric plexus, nestled between the longitudinal and circular smooth muscle layers, is chiefly responsible for orchestrating complex patterns of smooth muscle contraction/relaxation to regulate peristalsis. In contrast, the submucosal plexus, positioned between the circular smooth muscle layer and the mucosal lamina propria, has the unique capability to interact with epithelial cells to regulate selective permeability. That said, both plexuses share a similar ability to functionally interact with the gut-resident immune system (Progatzky and Pachnis, 2022; Yoo and Mazmanian, 2017).

Current state of knowledge strongly suggests that many roles of the ENS are in fact mainly fulfilled by EGCs, rather than enteric neurons (Boesmans et al., 2021; Lefevre et al., 2023; Seguella and Gulbransen, 2021; Rosenberg and Rao, 2021). Notable examples include the regulation of gastrointestinal immunity (Progatzky et al., 2021) and epithelial integrity (Baghdadi et al., 2022), where EGCs can detect damages and release appropriate pro-resolution substances (Progatzky and Pachnis, 2022; Lefevre et al., 2023; Seguella and Gulbransen, 2021; Baghdadi and Kim, 2023). In addition, EGCs are not only essential for providing trophic support to enteric neurons, but even have the potential to differentiate into enteric neurons themselves (Belkind-Gerson et al., 2017; Joseph et al., 2011; Laranjeira et al., 2011; McCallum et al., 2020). A prevalent view in the field is that all these different roles played by EGCs are distributed across different EGC subtypes. However, there is currently no clear consensus on how to classify these EGC subtypes. Depending on the criteria used, between three to nine subtypes of EGCs have been described (Lefevre et al., 2023). The more widely used classification system is based on topo-morphological criteria established from the study of adult rodents (Boesmans et al., 2015; Gulbransen and Sharkey, 2012; Charrier and Pilon, 2017; Hanani and Reichenbach, 1994), also validated in zebrafish (McCallum et al., 2020). This classification system allows to categorize EGCs into four main subtypes, each with a specific morphology linked to a particular location: highly branched Type I in ganglia, fibrous Type II in interganglionic fibers, multipolar unbranched Type III outside ganglia and interganglionic fibers, and bipolar Type IV deeper in muscles. Yet, how these (or any other) EGC subtypes are generated is currently elusive.

An important limitation for the study of EGC diversity is that we do not really know when diversification occurs. The early postnatal period is presumably critical, when both plexuses of the ENS must adapt to structural changes in the bowel wall. These structural changes might be a driving force for EGC diversification, at least based on topological criteria. Indeed, both the size and thickness of the bowel undergo significant changes during this period, implying that both plexuses must expand and transform in a coordinated manner to ensure various vital gastrointestinal functions (Roberts et al., 2007). In the myenteric plexus, this is notably reflected by the morphological transformation from dense juxtaposed groups of cells into individual and sparse ganglia, a process also accompanied by the expansion of both glial (Cossais et al., 2016) and neuronal (Liu et al., 2009) cell populations as well as by changes in the proportion of cholinergic and nitrergic neuron subtypes (de Vries et al., 2010). Although much less studied, similar maturation-associated changes have also been reported in the submucosal plexus (Young and Ciampoli, 1998).

Another potential source of EGC diversity might reside in the different origins of ENS progenitors, which are mainly derived either directly from neural crest cells or indirectly via neural crest-derived Schwann cell precursor (SCPs) (Pilon, 2021). The contribution of SCPs to the pool of enteric neurons is relatively well documented, with multiple reports supporting the existence of SCP-derived neurons in evolutionary distant species like the lamprey (Green et al., 2017), chicken (Espinosa-Medina et al., 2017), zebrafish (El-Nachef and Bronner, 2020; Kuil et al., 2023), mouse (Soret et al., 2020; Uesaka et al., 2015; Uesaka et al., 2021) and human (Woods et al., 2021). In contrast, although studies in both normal (Uesaka et al., 2015) and ENS-deficient (Soret et al., 2020; Uesaka et al., 2021) mice strongly suggest that SCPs can give rise to EGCs as well, this contribution has not been systematically assessed so far. In particular, the possibility that SCPs preferentially contribute to certain subtypes of EGCs, as they do for the Calretinin-positive subtype of enteric neurons in healthy mice (Uesaka et al., 2015), is unknown.

In the current study, we carefully analyzed the emergence of EGC diversity from a spatiotemporal perspective in two segments of the murine gastrointestinal tract (distal ileum and distal colon), while also assessing the specific contribution of SCPs to the EGC pool. Our immunofluorescence analyses revealed asynchronous emergence of the four main topo-morphological EGC subtypes during the early postnatal period, with different order of appearance along the radial axis (myenteric plexus vs. submucosal plexus). Our genetic cell lineage tracing experiments further showed a greater contribution of SCPs to the EGC pool in the colon than in the ileum, as previously reported for enteric neurons. Most interestingly, this contribution was also found not to be equal among the four topo-morphological subtypes, with a bias for Type IV in the myenteric plexus / circular muscle and a bias for Type II in the submucosal plexus. These findings provide a stepping stone for a better understanding of EGC diversification, thereby also opening new avenues for exploring functional diversity within the ENS.

## 2 Materials and methods

### 2.1 Mice

Wild-type FVB mice [FVB/NCrl; Strain code 207] were obtained from Charles River Laboratory, whereas the *Dhh*-Cre line [Jax stock #012929; FVB*(Cg)-Tg(Dhh-cre)1Mejr/J*] was obtained from the Jackson Laboratory. The *Rosa26*^[*FloxedSTOP*]*YFP*^ line [*Gt(ROSA)26Sor*^*TM*1*(EYFP)Cos*]^ was kindly directly provided by Dr. Frank Costantini (Columbia University). Both *Dhh*-Cre and *Rosa26*^[*FloxedSTOP*]*YFP*^ mice were maintained on the FVB background and genotyped by standard PCR using primers listed in Supplementary Table 1. Breeding couples were kept in individually ventilated cages and fed Charles River Rodent Diet #5075 (Cargill Animal *Nutrition*). All experiments were conducted in accordance with the guidelines of the Canadian Council on Animal Care (CCAC) and approved by the institutional committee (CIPA #992) of the Université du Québec à Montreal (UQAM). Euthanasia was performed either by decapitation for mice aged between postnatal day (P) 1 to P10, or by exposure to carbon dioxide (CO_2_) following isoflurane anesthesia for P20 mice. Animals from both sexes were included for each time point (see supplemental Data Sheet 1 for more details).

### 2.2 Sample collection and processing

After euthanasia, mice were pinned to a styrofoam slab to facilitate the removal of the skin and peritoneum, providing access to the gastrointestinal system. The small intestine and colon were then collected and kept in ice-cold 1X PBS (Phosphate-Buffered Saline) throughout the duration of the dissection. For both segments, we used only the most distal quarter, either just upstream of the cecum (distal ileum) or the anus (distal colon). Each sample was cut longitudinally along the mesentery, extensively washed in 1X PBS to remove stool, and pinned with mucosa side up onto Sylgard-coated (Dow Corning, Freeland, MI) Petri dishes. Pinned tissues were subsequently fixed overnight in 4% paraformaldehyde diluted in 1X PBS, followed by three washes of 10 min in 1X PBS. Tissues from P20 mice were further carefully microdissected to peel off the mucosal layer (including the submucosal plexus) while also preserving the muscle layers (including the myenteric plexus). All samples were stored in 1X PBS at 4°C prior to whole-mount immunofluorescence staining.

### 2.3 Whole-mount immunofluorescence staining

Ileum and colon samples were initially permeabilized for 2 h in blocking solution (5% Fetal Bovine Serum and 1% Triton-X100 in 1X PBS), followed by overnight incubation at 4°C with primary antibodies diluted in same blocking solution. After three washes of 10 min in 1X PBS, samples were then incubated with relevant secondary antibodies diluted in blocking solution for 1 h 30, followed by three additional washes of 10 min in 1X PBS. During the second wash, samples were counterstained with DAPI (4’,6-diamidino-2-phenylindole) diluted in 1X PBS. The specific antibodies used, and their dilution factors are listed in Supplementary Table 2. To enable the observation of plexuses via confocal microscopy, the stained tissues were mounted between two 24 x 50 mm glass coverslips in 100% Glycerol.

### 2.4 Imaging and data analysis

All immunofluorescence images were acquired using a Nikon A1 confocal microscope with Plan Fluor 20x/0.75 MImm and Plan Apo λ 60x/1.40 objectives. For each biological replicate, between 3 and 10 representative z-stack images were acquired, and then analyzed using the ImageJ software. EGC subtypes were manually quantified using the “Cell Counter” plugin, based on the location of SOX10+ cells and/or morphology of S100β+ cells (Supplementary Figures 1–3 and Supplementary Movies 1, 2). EGC proportions were calculated by including all relevant EGC subtypes in the total count (Type I to IV in the myenteric plexus / circular muscle layer; Type I to III in the submucosal plexus). The surface area of ganglia, extraganglionic space and interganglionic fibers were manually delineated and measured using the “freehand selections” tool, as shown in Supplementary Figure 1C.

### 2.5 Statistics

Each experiment was performed using 3–4 biological replicates, and either 3–5 (for myenteric plexus / circular muscle) or 6–10 (for submucosal plexus) fields of view per replicate (see supplemental Data Sheet 1 for detailed information about each replicate, including sex and number of counted cells). For quantitative analysis, data are expressed as the mean ± standard deviation (SD), with the value of each imaging field also represented by a dot. Based on these single values, statistical significance of differences between groups was determined in GraphPad Prism 9.5.1, using either two-tailed Welch’s *t-*tests (when comparing two groups) or One-Way/Two-Way ANOVA with Tukey’s multiple comparison tests (when comparing more than two groups). Tests used are indicated in figure legends. Data were considered statistically significant when the *P*-value was less than 0.05.

## 3 Results

### 3.1 Topo-morphological subtypes of EGCs emerge sequentially during postnatal maturation of the myenteric plexus

To gain insight about the process of EGC diversification, we first analyzed the appearance of the four main topo-morphological subtypes of EGCs in the myenteric plexus and circular muscle of wild-type FVB mice. Using whole-mount immunostaining, we studied four time points (P1, P5, P10 and P20) spanning the postnatal period before weaning. The longitudinal muscle layer was excluded from this analysis because of interference from the juxtaposed serosa, which gave high background signal in immunofluorescence staining. Staining of EGCs and neuronal networks was performed using antibodies against SOX10 and βIII-Tubulin, respectively. SOX10 staining is great for cell counting, being also generally sufficient for identifying EGC subtypes based on topological criteria only (Supplementary Figure 1A and Supplementary Movies 1, 2)−as the unique morphology of each subtype is linked to a specific location, along both the x/y plane and the z-axis (Boesmans et al., 2015; Gulbransen and Sharkey, 2012; Charrier and Pilon, 2017; Hanani and Reichenbach, 1994). Notably, location along the z-axis is especially useful for distinguishing Type III from Type IV EGCs, as both subtypes are clearly not in the same plane (Supplementary Figure 1A and Supplementary Movies 1, 2). That said, we also co-stained all tissues for S100β to enable definitive identification of EGC subtypes based on gross morphological criteria as well. Although global S100β staining is not as good as sparce labeling approaches for revealing morphological details of EGCs (Boesmans et al., 2015; Hanani and Reichenbach, 1994; Rao et al., 2015), it nonetheless often proved to be useful for distinguishing between multipolar Type I and elongated Type II EGCs (from P5 onwards), when Type II EGCs are at the border of ganglia and oriented toward interganglionic fibers (Supplementary Figure 1B). Interestingly, our S100β staining data further revealed that each EGC subtype within the myenteric plexus / circular muscle gradually acquires its definitive gross morphological characteristics between P1 and P20 (Supplementary Figure 2). For the sake of simplicity, detailed S100β data are however not shown in the main figures. Given that as little as 2–6% of all SOX10+ cells from the myenteric plexus / circular muscle are negative for S100β during the investigated period (Table 1), we reasoned that the impact of this decision on our estimates of the relative proportions of EGC subtypes would be minimal.

**TABLE 1:** Percentage overlap between SOX10 and S100β stainings,

	$\frac{\mathrm{SOX10}+\mathrm{S100}\,\beta +\mathrm{cells}}{\mathrm{total}\,\mathrm{SOX10}+\mathrm{cells}}\left(\%\right)$			
	Ileum MP	Ileum SMP	Colon MP	Colon SMP
P1	93.5 ± 4.2	83.2 ± 12.4	94.0 ± 1.7	78.8 ± 11.9
P20	97.0 ± 3.5	96.2 ± 6.2	97.9 ± 1.8	97.5 ± 3.9

**FIGURE 1:**
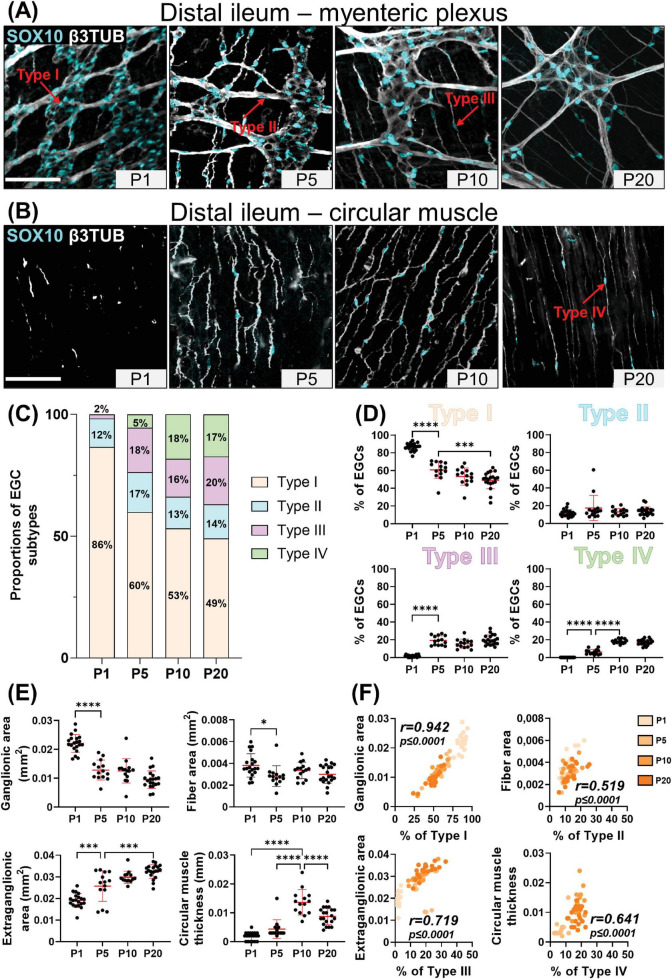
Analysis of EGC diversity in the maturing postnatal myenteric plexus of the distal ileum. **(A,B)** Immunofluorescence analysis of the myenteric plexus **(A)** and associated circular muscle **(B)** in the distal ileum of wild-type FVB mice, at indicated postnatal ages (P1, P5, P10 and P20). Intestinal tissues were immunolabeled with antibodies against SOX10 for EGCs (cyan) and βIII-Tubulin for neuronal fibers (gray). Displayed images are z-stack projections representative of observations made from *N* = 3 mice per time point. Scale bar, 70 μm. **(C,D)** Quantitative analysis of the relative proportions of EGC Type I to IV, using images such as those displayed in panels **(A,B)** (*N* = 3 mice per time point; *n* = 3 to 5 60x fields of view per animal). **(E)** Quantitative analysis of indicated morphometric parameters (ganglionic surface area, extraganglionic surface area, interganglionic fiber surface area and circular muscle thickness) in the distal ileum as a function of age during the early postnatal period. **(F)** Correlation analysis (excluding 0% and 100% values; incompatible with proportion calculations) between Type I proportion and ganglionic area, Type II proportion and interganglionic fiber area, Type III proportion and extraganglionic area, and Type IV and circular muscle thickness. Colored symbols correspond to the indicated time points. *r* is Pearson’s correlation coefficient. *P ≤ 0.05, ***P ≤ 0.001, ****P ≤ 0.0001; One-Way ANOVA and Tukey’s multiple comparison test.

**FIGURE 2:**
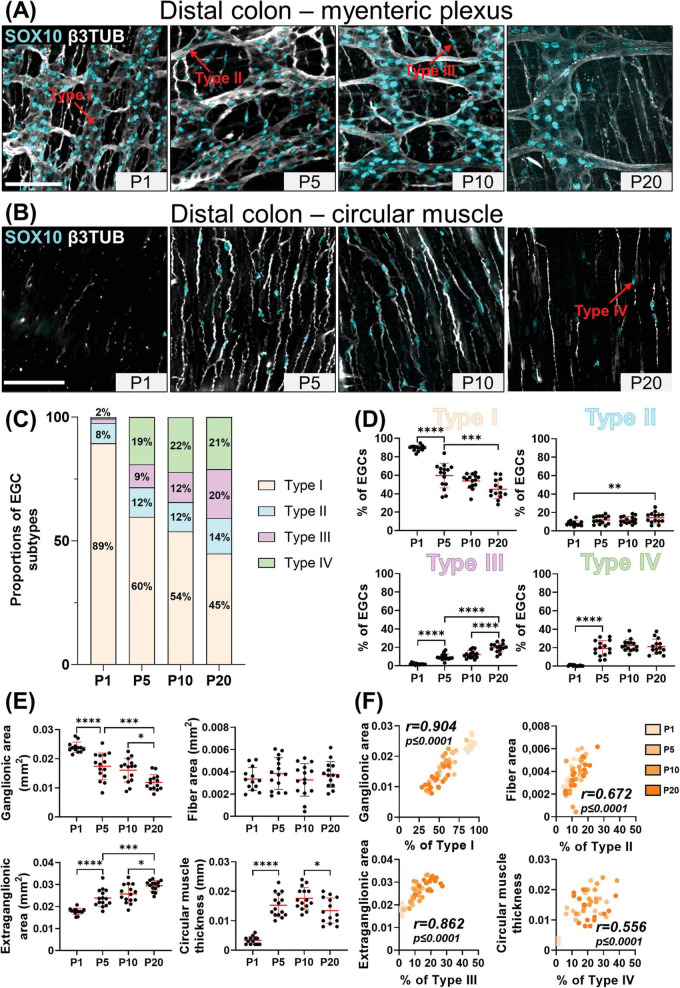
Analysis of EGC diversity in the maturing postnatal myenteric plexus of the distal colon. **(A,B)** Immunofluorescence analysis of the myenteric plexus **(A)** and associated circular muscle **(B)** in the distal colon of wild-type FVB mice, at indicated postnatal ages (P1, P5, P10 and P20). Intestinal tissues were immunolabeled with antibodies against SOX10 for EGCs (cyan) and βIII-Tubulin for neuronal fibers (gray). Displayed images are z-stack projections representative of observations made from N = 3 mice per time point. Scale bar, 70μm. **(C,D)** Quantitative analysis of the relative proportions of EGC Type I to IV, using images such as those displayed in panels **(A,B)** (*N* = 3 mice per time point; *n* = 3 to 5 60x fields of view per animal). **(E)** Quantitative analysis of indicated morphometric parameters (ganglionic surface area, extraganglionic surface area, interganglionic fiber surface area and circular muscle thickness) in the distal colon as a function of age during the early postnatal period. **(F)** Correlation analysis (excluding 0% and 100% values; incompatible with proportion calculations) between Type I proportion and ganglionic area, Type II proportion and interganglionic fiber area, Type III proportion and extraganglionic area, and Type IV and circular muscle thickness. Colored symbols correspond to the indicated time points. *r* is Pearson’s correlation coefficient. *P ≤ 0.05, **P ≤ 0.01, ***P ≤ 0.001, ****P ≤ 0.0001; One-Way ANOVA and Tukey’s multiple comparison test.

At P1, the vast majority of SOX10+ cells (> 85%) can be classified as Type I EGCs, in both the distal ileum (Figures 1A–D) and the distal colon (Figures 2A–D). Most of the remaining EGCs fall into the Type II category (8–12%), while the other EGC subtypes are either marginally detected (2% for Type III) or absent (Type IV) in both bowel segments at this stage (Figures 1A–D, 2A–D). At P5, the proportions of Type III and Type IV EGCs are both markedly increased, with a steeper rise in Type III in the distal ileum (Figures 1A–D) and Type IV in the distal colon (Figures 2A–D). These increased proportions of Type III and Type IV are mostly made at the expense of Type I EGCs, while the proportion of Type II EGCs remains relatively stable in both bowel segments (Figures 1A–D, 2A–D). The pool of Type III and Type IV EGCs continue to grow at P10 and P20, with asymmetric patterns favoring Type IV in the distal ileum (Figures 1A–D) or Type III in the distal colon (Figures 2A–D). As the proportion of Type II remains stable at all analyzed time points, this expansion of the pool of Type III and Type IV EGCs continues to be made at the expense of Type I EGCs in both bowel segments. Nonetheless, Type I EGCs are still the most common subtype, representing about half (49 ± 10% in distal ileum; 45 ± 10% in distal colon) of all EGCs at P20 (Figures 1A–D, 2A–D). At this stage, Type III (20 ± 6% in distal ileum; 20 ± 5% in distal colon) and Type IV (17 ± 3% in distal ileum; 21 ± 8% in distal colon) EGCs are now equally represented, both outnumbering Type II EGCs (14 ± 5% in distal ileum; 14 ± 6% in distal colon) (Figures 1A–D, 2A–D).

We next verified the possibility that the sequential appearance of the four topo-morphological subtypes of EGCs described above could be related to structural changes that occur in the bowel wall between P1 and P20. To this end, we carefully measured the surface area of ganglia, extraganglionic space, and interganglionic fibers as well as the thickness of the circular muscle layer (Supplementary Figure 1C), in all samples used for EGC subtype quantification in both the distal ileum (Figure 1E) and distal colon (Figure 2E). In accordance with the observed loosening of the connections between myenteric ganglia in both bowel segments, these analyses revealed a significant stage-dependant decrease in ganglionic surface area accompanied by a commensurate increase in extraganglionic surface area (Figures 1E, 2E). When analyzed as a function of EGC subtype proportions in corresponding samples, these structural changes are strongly correlated with the proportions of Type I EGCs and Type III EGCs, respectively (Figures 1F, 2F). The surface area of interganglionic fibers was not found to vary much overall between P1 and P20 (Figures 1E, 2E), similar to the proportion of Type II EGCs (Figures 1D, 2D), but both parameters are nonetheless again positively correlated when analyzed as a function of each other in each sample (Figures 1F, 2F). Lastly, we noted that the thickness of the circular muscle layer generally increases with age in both bowel segments, peaking at P10 and then slightly decreasing at P20. However, this increase was found to be delayed in the distal ileum compared to the distal colon, being suddenly detectable at P10 and P5, respectively (Figures 1E, 2E). Remarkably, this particular kinetics mimics the abrupt increase in the proportion of Type IV EGCs detected in each bowel segment, hence resulting in a positive correlation once again (Figures 1F, 2F).

From this systematic characterization of the maturing myenteric plexus / circular muscle, we can thus conclude that the four topo-morphological subtypes of EGCs appear in numerical order, from Type I/II (co-occurring) to Type IV, regardless of the bowel segment examined. Moreover, our correlation data strongly support the notion that structural changes in the early postnatal bowel, including within the ENS, have a profound influence on the relative proportions of these four EGC subtypes.

### 3.2 Topo-morphological subtypes of EGCs emerge sequentially during postnatal maturation of the submucosal plexus, but in a different order compared to the myenteric plexus

To complement our analyses of the myenteric plexus / circular muscle, we then turned to the submucosal plexus (largely excluding the mucosa), using the exact same experimental design as described above. Here, DAPI staining (in conjunction with βIII-Tubulin staining) proved to be useful for delineating the tiny submucosal ganglia, based on the round nucleus of neurons (Supplementary Figure 3). We also found that the overlap between SOX10 and S100β stainings is much lower here than in the myenteric plexus at P1 (Table 1), but we validated that proportions of EGC subtypes are nonetheless very similar when using combined SOX10-S100β data instead of SOX10 data alone at both P1 (ileum) and P5 (colon) (Supplementary Figure 4). Of note, S100β data further revealed that acquisition of definitive gross morphological attributes is delayed in the submucosal plexus (Supplementary Figure 2B) compared to the myenteric plexus (Supplementary Figure 2A).

**FIGURE 3:**
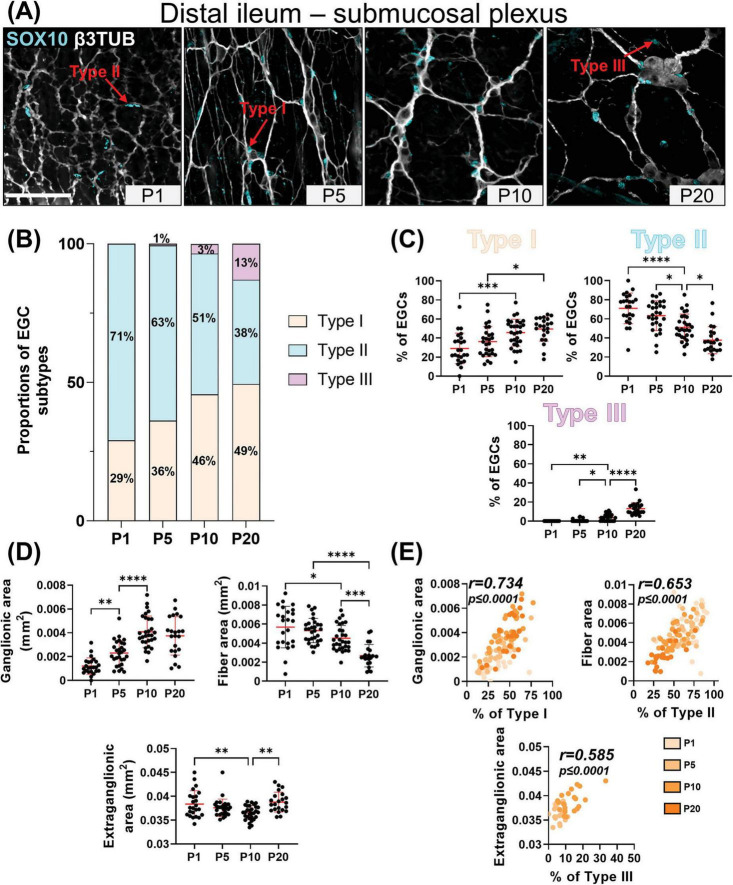
Analysis of EGC diversity in the maturing postnatal submucosal plexus of the distal ileum. **(A)** Immunofluorescence analysis of the submucosal plexus in the distal ileum of wild-type FVB mice, at indicated postnatal ages (P1, P5, P10 and P20). Intestinal tissues were immunolabeled with antibodies against SOX10 for EGCs (cyan) and βIII-Tubulin for neuronal fibers (gray). Displayed images are z-stack projections representative of observations made from *N* = 3 mice per time point. Scale bar, 70 μm. **(B,C)** Quantitative analysis of the relative proportions of EGC Type I to IV, using images such as those displayed in panel A (N = 3 mice per time point; *n* = 3 to 5 60x fields of view per animal). **(D)** Quantitative analysis of indicated morphometric parameters (ganglionic surface area, extraganglionic surface area, and interganglionic fiber surface area) in the distal ileum as a function of age during the early postnatal period. **(E)** Correlation analysis (excluding 0% and 100% values; incompatible with proportion calculations) between Type I proportion and ganglionic area, Type II proportion and interganglionic fiber area, and Type III proportion and extraganglionic area. Colored symbols correspond to the indicated time points. *r* is Pearson’s correlation coefficient. *P ≤ 0.05, **P ≤ 0.01, ***P ≤ 0.001, ****P ≤ 0.0001; One-Way ANOVA and Tukey’s multiple comparison test.

**FIGURE 4:**
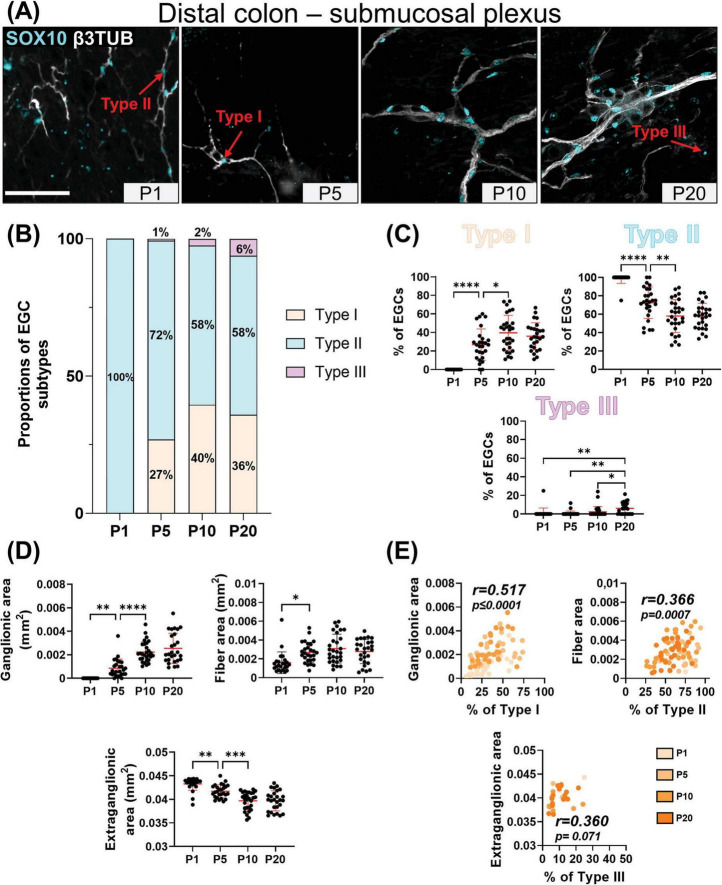
Analysis of EGC diversity in the maturing postnatal submucosal plexus of the distal colon. **(A)** Immunofluorescence analysis of the submucosal plexus in the distal colon of FVB WT mice, at indicated postnatal ages (P1, P5, P10 and P20). Intestinal tissues were immunolabeled with antibodies against SOX10 for EGCs (cyan) and βIII-Tubulin for neuronal fibers (gray). Displayed images are z-stack projections representative of observations made from N = 3 mice per time point. Scale bar, 70μm. **(B-C)** Quantitative analysis of the relative proportions of EGC Type I to IV, using images such as those displayed in panel A (N = 3 mice per time point; n = 3 to 5 60x fields of view per animal). **(D)** Quantitative analysis of indicated morphometric parameters (ganglionic surface area, extraganglionic surface area, and interganglionic fiber surface area) in the distal colon as a function of age during the early postnatal period. **(E)** Correlation analysis (excluding 0% and 100% values; incompatible with proportion calculations) between Type I proportion and ganglionic area, Type II proportion and interganglionic fiber area, and Type III proportion and extraganglionic area. Colored symbols correspond to the indicated time points. *r* is Pearson’s correlation coefficient. *P ≤ 0.05, **P ≤ 0.01, ***P ≤ 0.001, ****P ≤ 0.0001; One-Way ANOVA and Tukey’s multiple comparison test.

Our detailed SOX10 immunofluorescence analyses again revealed the sequential appearance of EGC subtypes between P1 and P20, but not in the same order as in the myenteric plexus and with more pronounced regional differences between distal ileum (Figures 3A–C) and distal colon (Figures 4A–C). At P1, Type II EGCs are clearly predominating in the submucosal plexus, being even virtually exclusive in the distal colon (Figures 4A–C). This pattern is inversely reflected in the detection of Type I EGCs, which are present in the distal ileum at P1 (Figures 3A–C) but are not detected until P5 in the distal colon (Figures 4A–C). This population of Type I EGCs then generally grows over time in both bowel segments (Figures 3A–C, 4A–C), until eventually outnumbering Type II EGCs in the distal ileum at P20 (49 ± 13% of Type I *vs* 38 ± 14% of Type II), but not in the distal colon where Type II remains the most common subtype at this stage as well (36 ± 15% of Type I *vs* 58 ± 14% of Type II). In both bowel segments (Figures 3A–C, 4A–C), Type III EGCs are not detected at P1, then forming a relatively marginal population until P20, at which stage they are more numerous in the distal ileum (13 ± 6%) than in the distal colon (6% ± 6%). However, it is important to bear in mind that these proportions only take into account Type III EGCs close to the submucosal plexus (a.k.a. Type III_[SMP]_), and not those located deeper in villi (a.k.a. Type III_[mucosa]_), which are difficult to visualize along the z-axis after whole-mount staining (as performed in the current study).

Comparison with morphometric parameters of the submucosal plexus again revealed a robust correlation between the surface area occupied by ENS ganglia and the proportion of Type I EGCs, in each sample from the distal ileum (Figures 3D–E) and distal colon (Figures 4D–E). Yet, at the difference of the myenteric plexus, where the ganglionic surface area decreases between P1 and P20 in both bowel segments (Figures 1E, 2E), the corresponding surface area in the submucosal plexus was found to generally increase over time (Figures 3D, 4D) – in line with the notion that the submucosal plexus initially develops via inward cell migration from the myenteric plexus (McKeown et al., 2001; Singh et al., 2015; Lasrado et al., 2017). Accordingly, the surface area of the extraganglionic space, which markedly increases over time in the myenteric plexus (Figures 1E, 2E), was here found to slightly decrease in the submucosal plexus from P1 onwards (Figures 3D, 4D). Despite this difference between plexuses, a positive correlation can again be observed when the extraganglionic surface area is analyzed as a function of the proportion of Type III_[SMP]_ EGCs in each sample from both bowel segments (Figures 3E, 4E). This correlation is however weaker in the distal colon, where fewer Type III_[SMP]_ EGCs were detected. A more striking regional difference was noted for the surface area occupied by interganglionic submucosal nerves between P1 and P20, resulting in a robust stage-dependent decrease in the distal ileum (Figure 3D) and, conversely, a modest stage-dependent increase in the distal colon (Figure 4D). Nonetheless, a positive correlation with the proportion of Type II EGCs can once again be noted in both bowel segments (Figures 3E, 4E), this correlation being again weaker in the distal colon.

In sum, our immunofluorescence data indicate that EGC diversification based on topo-morphological criteria takes a different path in the submucosal plexus, with Type II EGCs appearing first, followed by Type I and Type III, respectively. Also, our correlation data again support a model whereby EGC diversification is generally impacted by structural changes in the ENS.

### 3.3 SCPs preferentially contribute to specific topo-morphological EGC subtypes, which vary depending of bowel segment

**FIGURE 5:**
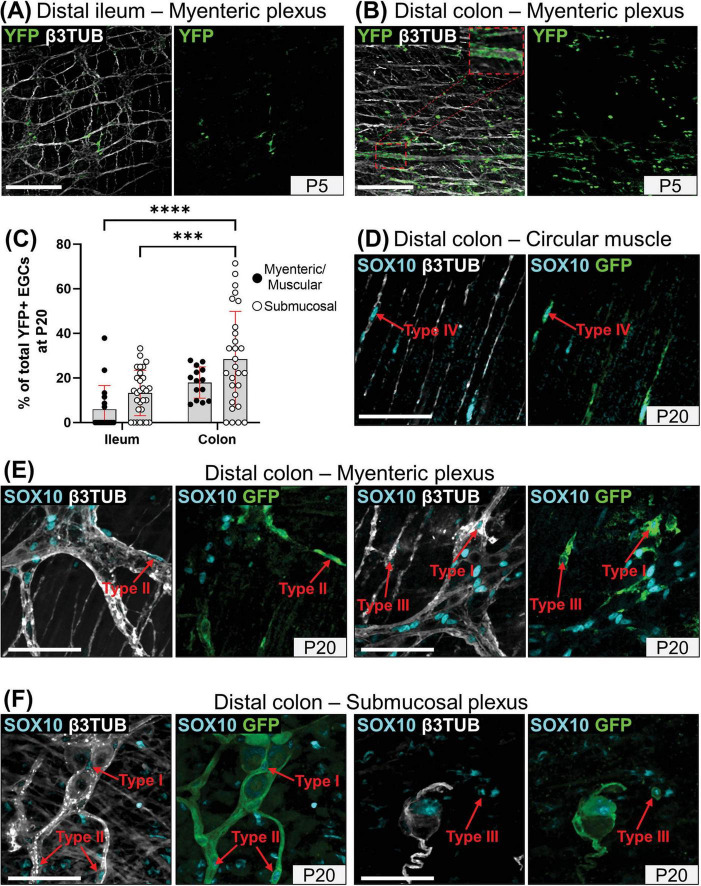
Global analysis of the SCP contribution to enteric gliogenesis. **(A,B)** Immunofluorescence-based analysis of the distribution of YFP+ SCPs in the myenteric plexus of the distal ileum **(A)** and distal colon **(B)** from *Dhh*-Cre;*Rosa26*^[FloxedSTOP]YFP^ mice at P5. Intestinal tissues were immunolabeled with antibodies against GFP/YFP for SCP-derived cells (green) and βIII-Tubulin for neuronal fibers (grey). Displayed images are z-stack projections representative of observations made from N = 3 mice per time point. Scale bar, 200 μm. **(C)** Quantitative analysis of the global SCP contribution to enteric gliogenesis in the myenteric plexus/muscular layer (black dots) and submucosal plexus (white dots) of the distal ileum and distal colon from *Dhh*-Cre;*Rosa26*^[FloxedSTOP]YFP^ mice at P20. Each dot represents the percentage of YFP+ SOX10+ EGCs over the total of SOX10+ EGCs in a single 60x field of view for *N* = 3 mice (*n* = 3–5 fields of view per tissue for the myenteric plexus/muscular layer; *n* = 6–10 fields of view per tissue for the submucosal layer). **(D-F)** Immunofluorescence analysis of the circular muscle layer **(D)**, the myenteric plexus **(E)** and the submucosal plexus **(F)** of the distal colon from *Dhh*-Cre;*Rosa26*^[FloxedSTOP]YFP^ mice at P20. Tissues were immunolabeled with SOX10 (cyan), GFP/YFP (green) and βIII-Tubulin antibodies (gray). Scale bar, 70 μm. ***P ≤ 0.001, ****P ≤ 0.0001; Two-Way ANOVA and Tukey’s multiple comparison test.

To evaluate the specific contribution of SCPs to enteric gliogenesis during early postnatal development, we used the same genetic cell lineage tracing system as previously used to identify SCP derivatives in the bowel (Soret et al., 2020) and the inner ear (Bonnamour et al., 2021), comprising the transgenic *Dhh-Cre* driver (Jaegle et al., 2003) and the *Rosa26*^[FloxedSTOP]YFP^ reporter allele (Srinivas et al., 2001). When ENS progenitors directly derived from neural crest cells have just completed their colonization of the developing bowel (i.e., at embryonic day 14.5), this system specifically labels a subset of SOX10+ SCPs on mesentery-associated nerves, and none of the SOX10+ ENS cells already present in the ileum and colon (Supplementary Figure 5). A similar conclusion was previously drawn using the same Cre driver with a different Cre reporter allele, and this study further reported that SCPs start to colonize the developing bowel a few days later, just before birth (Uesaka et al., 2015). As also reported in this prior work focused on enteric neurogenesis (Uesaka et al., 2015), our detailed immunofluorescence analyses revealed that the contribution of YFP+ SCPs to the general pool of ENS cells after birth is much greater in the colon than in the ileum, at all stages examined between P1 and P20. As evidenced in the myenteric plexus at P5 (Figures 5A, B) we further found that this contribution is unevenly distributed, being apparently more extensive in the vicinity of extrinsic nerves that constitute the entry point for SCPs (see inset in Figure 5B). This patchy distribution led to widely distributed values in our global quantitative analysis of SCP-derived EGCs at P20 (Figure 5C). Interestingly, this analysis nonetheless highlighted the fact that the SCP contribution is also generally lower in the myenteric plexus/circular muscle than in the submucosal plexus, in both bowel segments (Figure 5C).

**FIGURE 6:**
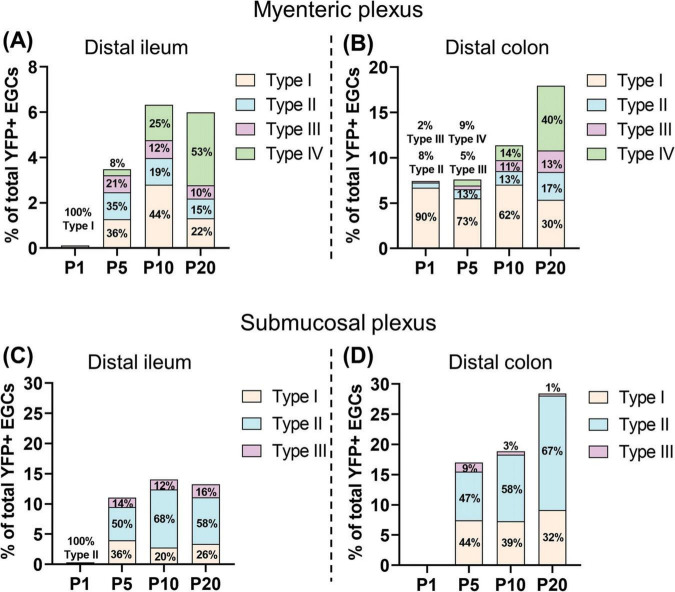
Detailed analysis of the SCP contribution to EGC diversity. **(A–D)** Quantitative analysis of the SCP contribution to enteric gliogenesis as a function of EGC Types I to IV, in the myenteric plexus/muscular layer of the distal ileum **(A)** and the distal colon **(B)**, as well as in the submucosal plexus of the distal ileum **(C)** and the distal colon **(D)**. EGC subtypes were quantified using images such as those displayed in Figures 5D–F (*N* = 3 mice; n = 3–5 fields of view per tissue for the myenteric plexus/muscular layer; *n* = 6–10 fields of view per tissue for the submucosal layer). Size of sub-bars corresponds to the average of the percentage of YFP+ SOX10+ EGCs among all SOX10+ EGCs, in the indicated tissue layers and bowel segments.

When analyzed as a function of topo-morphological subtypes, our immunofluorescence data then revealed that SCPs can contribute to every EGC subtype from both plexuses, as notably shown in the colon at P20 (Figures 5D–F). However, it is important to note that the final SCP contribution at P20 is clearly biased to particular EGC subtypes in a plexus-specific manner, favoring EGC Type IV in the myenteric plexus / circular muscle (40–53% of all SCP-derived EGCs; Figures 6A, B and Supplementary Figures 6A, B) and Type II in the submucosal plexus (58–67% of all SCP-derived EGCs; Figures 6C, D and Supplementary Figures 6C, D), in both bowel segments. Each bias seems to be established in accordance with the kinetics of appearance of their corresponding EGC subtype between P1 and P20, with the Type IV bias in the myenteric plexus / circular muscle (see Figures 6A, B *vs.* Figures 1C, 2C) appearing in a delayed manner relative to the Type II bias in the submucosal plexus (see Figures 6C, D *vs.* Figures 3C, 4C). Type I EGCs in the myenteric plexus / circular muscle from the distal colon initially constitute the highest contribution of SCPs, but this contribution then markedly decreases to ultimately stabilize around 26–32% in all locations at P20 (Figure 6 and Supplementary Figure 6). Type III EGCs constitute the lowest contribution overall, representing only 1–16% of SCP-derived EGCs at P20 (Figure 6 and Supplementary Figure 6). In conclusion, the SCP contribution to the EGC pool is not equal, being regionally different according to both the longitudinal (ileum *vs*. colon) and the radial (myenteric plexus / muscle *vs.* submucosal plexus) axes of the bowel.

## 4 Discussion

In this study, we investigated EGC diversification during the postnatal maturation of the myenteric and submucosal plexuses in both the distal ileum and distal colon. Combined with cell lineage tracing studies, our quantitative analyses highlighted important regional differences in the proportions and origins of the different topo-morphological subtypes of EGCs. That said, it is necessary to interpret these relative proportions for what they are, that is an estimate – not definitive exact values. Using SOX10 staining data alone had the potential disadvantage of including postnatal ENS progenitors in our calculations (Bondurand et al., 2003), although this possibility must also be considered with caution as the distinction between *bona fide* ENS progenitors and neurogenic EGCs becomes increasingly blurred (Guyer et al., 2023). The alternative of systematically using combined SOX10-S100β staining data for these calculations might have yielded slightly different proportions (Supplementary Figure 4), but these modest differences would not have changed our general conclusions. Therefore, regardless of precise values, we expect that the new insights provided by our work will pave the way for more comprehensive studies of the ENS in health and disease, as discussed below.

At P20 (*i.e.*, at the end of postnatal maturation), there is no difference between the distal ileum and distal colon in terms of proportions of the four topo-morphological EGC subtypes in the myenteric plexus / circular muscle, with Type I EGCs clearly predominating in both segments. Yet, a difference was noted in the kinetics of expansion of Type III and Type IV pools during earlier stages, with sudden increases of Type III in the ileum and Type IV in the colon, the proportions of both then remaining stable over time. More striking differences can be noted when comparing myenteric and submucosal plexuses in general, with Type II EGCs being much more frequent in the submucosal plexus. In this case, there is also a notable regional difference in that Type II EGCs even outnumber Type I EGCs specifically in the distal colon. When also integrated with the structural changes that occur in the bowel wall, all these complex spatiotemporal differences suggest that Type I and II EGCs from the myenteric plexus might give rise to the other topo-morphological subtypes in the myenteric plexus (via lateral and short radial migration) and submucosal plexus (via longer inward migration). Newcoming Type II EGCs in the submucosal plexus might then also contribute to generate EGC diversity as well, by acting as a local source of Type I and Type III EGCs. Testing these hierarchical models will be technically challenging in mice, although not impossible by combining genetic cell lineage tracing (using a tamoxifen-inducible *Sox10-Cre* driver for instance) (Deal et al., 2021) with live imaging of bowel explants. This question might in fact be easier to address in the more imaging-friendly zebrafish, in which all topo-morphological subtypes of EGCs have also been described (McCallum et al., 2020). An important related objective would be to identify and understand the signaling pathways involved in the directed migration of EGC subtypes. The Netrin/DCC pathway is a prime candidate for the control of radial migration (Jiang et al., 2003), but most likely not the only one. Knowing which pathways are involved and how they work would be especially useful for devising new or improved ENS therapies based on *in situ* stimulation with exogenous growth factors (Pilon, 2021; Soret et al., 2020; Soret et al., 2021) or transplantation of genetically modified ENS progenitors (Mueller et al., 2024).

Beyond spatiotemporal considerations, we found that the different types of ENS progenitors can also be a significant source of EGC diversity. Indeed, although SCPs have the potential to generate all topo-morphological subtypes of EGCs, they have a marked preference for differentiating into Type IV in the myenteric plexus / circular muscle and into Type II in the submucosal plexus. By extension, these data thus indicate that neural crest-derived ENS progenitors are by far the main source of Type I and Type III EGCs. This discovery is reminiscent of the biased origin of fibrous *vs* protoplasmic astrocytes in the central nervous system, where fibrous astrocytes are mainly derived from subventricular neural progenitors whereas protoplasmic astrocytes are rather preferentially derived from radial glia (Sovrea and Bosca, 2013). These different developmental pathways are believed to also impact the transcriptional signature and hence the function of astrocyte subtypes (Tabata, 2015). Whether this is also the case for SCP-derived EGCs will definitely be an exciting question to address in future work. In this regard, a previously published bulk RNA-seq analysis of *Plp1-GFP*+ EGCs suggests that molecular traces of an SCP origin might be present in some EGCs (Rao et al., 2015). However, a firm conclusion cannot be drawn without multi-marker staining of gastrointestinal tissues, and our preliminary investigations along these lines were not successful for *Mpz* – one of the candidate SCP marker gene identified in the previous bulk RNA-seq experiment (Rao et al., 2015). Using immunofluorescence, we failed to detect the corresponding protein in SCP-derived EGCs, while the same antibody works well for labeling sciatic nerve-associated Schwann cells (Supplementary Figure 7). This question of whether there is some kind of connection between topo-morphological and transcriptional subtypes of EGCs is certainly of great interest to the field (Lefevre et al., 2023), as notably highlighted in some studies recently posted as preprints (Majd et al., 2022; Windster et al., 2023). With the identification of subtype-specific markers will come the possibility to precisely evaluate the *in vivo* functional importance of specific EGC subtypes via Cre/Lox-induced cell ablation with diphteria toxin-expressing reporters, as previously done for larger populations of EGCs (Baghdadi et al., 2022; Rao et al., 2017).

Regardless of where future research leads us, the new knowledge gained from the current study is already particularly important for all studies dealing with ENS regeneration (Pilon, 2021). Any project in this regard should now consider verifying the proper re-establishment of EGC subtypes in complementary proportions, as this is most likely essential for the normal functioning of the regenerated ENS. Given that EGC diversification is greatly influenced by the origin of ENS progenitors, this is also a call for using mixtures of different types of ENS progenitors in cell transplantation-based approaches. Moreover, the fact that we now know the relative proportions of the different topo-morphological EGC subtypes during postnatal maturation of the ENS (in both the myenteric and the submucosal plexus from two bowel segments) will allow to verify the physiological status of the gastrointestinal tract in health and disease with a much higher degree of precision.

## Data Availability

The raw data supporting the conclusions of this article will be made available by the authors, without undue reservation.

## References

[B1] Baghdadi M. Kim T. (2023). The multiple roles of enteric glial cells in intestinal homeostasis and regeneration. *Semin. Cell Dev. Biol.* 150-151 43–49. 36658046 10.1016/j.semcdb.2023.01.005

[B2] Baghdadi M. Ayyaz A. Coquenlorge S. Chu B. Kumar S. Streutker C. (2022). Enteric glial cell heterogeneity regulates intestinal stem cell niches. *Cell Stem Cell* 29:86-100.e6. 10.1016/j.stem.2021.10.004 34727519

[B3] Belkind-Gerson J. Graham H. Reynolds J. Hotta R. Nagy N. Cheng L. (2017). Colitis promotes neuronal differentiation of Sox2+ and PLP1+ enteric cells. *Sci. Rep.* 7:2525. 10.1038/s41598-017-02890-y 28566702 PMC5451421

[B4] Boesmans W. Lasrado R. Vanden Berghe P. Pachnis V. (2015). Heterogeneity and phenotypic plasticity of glial cells in the mammalian enteric nervous system. *Glia* 63 229–241. 25161129 10.1002/glia.22746

[B5] Boesmans W. Nash A. Tasnady K. Yang W. Stamp L. Hao M. (2021). Development, diversity, and neurogenic capacity of enteric glia. *Front. Cell Dev. Biol.* 9:775102. 10.3389/fcell.2021.775102 35111752 PMC8801887

[B6] Bondurand N. Natarajan D. Thapar N. Atkins C. Pachnis V. (2003). Neuron and glia generating progenitors of the mammalian enteric nervous system isolated from foetal and postnatal gut cultures. *Development* 130 6387–6400. 10.1242/dev.00857 14623827

[B7] Bonnamour G. Soret R. Pilon N. (2021). Dhh-expressing Schwann cell precursors contribute to skin and cochlear melanocytes, but not to vestibular melanocytes. *Pigment Cell Melanoma Res.* 34 648–654. 10.1111/pcmr.12938 33089656

[B8] Charrier B. Pilon N. (2017). Toward a better understanding of enteric gliogenesis. *Neurogenesis* 4:e1293958. 10.1080/23262133.2017.1293958 28352645 PMC5358706

[B9] Cossais F. Durand T. Chevalier J. Boudaud M. Kermarrec L. Aubert P. (2016). Postnatal development of the myenteric glial network and its modulation by butyrate. *Am. J. Physiol. Gastrointest. Liver Physiol.* 310 G941–G951. 10.1152/ajpgi.00232.2015 27056724

[B10] de Vries P. Soret R. Suply E. Heloury Y. Neunlist M. (2010). Postnatal development of myenteric neurochemical phenotype and impact on neuromuscular transmission in the rat colon. *Am. J. Physiol. Gastrointest. Liver Physiol.* 299 G539–G547. 10.1152/ajpgi.00092.2010 20522637

[B11] Deal K. Rosebrock J. Eeds A. DeKeyser J. Musser M. Ireland S. (2021). Sox10-cre BAC transgenes reveal temporal restriction of mesenchymal cranial neural crest and identify glandular Sox10 expression. *Dev. Biol.* 471 119–137. 10.1016/j.ydbio.2020.12.006 33316258 PMC7855809

[B12] El-Nachef W. Bronner M. (2020). De novo enteric neurogenesis in post-embryonic zebrafish from Schwann cell precursors rather than resident cell types. *Development* 147:dev186619. 10.1242/dev.186619 32541008 PMC7375481

[B13] Espinosa-Medina I. Jevans B. Boismoreau F. Chettouh Z. Enomoto H. Muller T. (2017). Dual origin of enteric neurons in vagal Schwann cell precursors and the sympathetic neural crest. *Proc. Natl. Acad. Sci. U.S.A.* 114 11980–11985. 10.1073/pnas.1710308114 29078343 PMC5692562

[B14] Furness J. (2012). The enteric nervous system and neurogastroenterology. *Nat. Rev. Gastroenterol. Hepatol.* 9 286–294. 22392290 10.1038/nrgastro.2012.32

[B15] Green S. Uy B. Bronner M. (2017). Ancient evolutionary origin of vertebrate enteric neurons from trunk-derived neural crest. *Nature* 544 88–91. 10.1038/nature21679 28321127 PMC5383518

[B16] Gulbransen B. Sharkey K. (2012). Novel functional roles for enteric glia in the gastrointestinal tract. *Nat. Rev. Gastroenterol. Hepatol.* 9 625–632. 22890111 10.1038/nrgastro.2012.138

[B17] Guyer R. Stavely R. Robertson K. Bhave S. Mueller J. Picard N. (2023). Single-cell multiome sequencing clarifies enteric glial diversity and identifies an intraganglionic population poised for neurogenesis. *Cell Rep.* 42:112194. 10.1016/j.celrep.2023.112194 36857184 PMC10123761

[B18] Hanani M. Reichenbach A. (1994). Morphology of horseradish peroxidase (HRP)-injected glial cells in the myenteric plexus of the guinea-pig. *Cell Tissue Res.* 278 153–160. 7954696 10.1007/BF00305787

[B19] Jaegle M. Ghazvini M. Mandemakers W. Piirsoo M. Driegen S. Levavasseur F. (2003). The POU proteins Brn-2 and Oct-6 share important functions in Schwann cell development. *Genes Dev.* 17 1380–1391. 10.1101/gad.258203 12782656 PMC196070

[B20] Jiang Y. Liu M. Gershon M. (2003). Netrins and DCC in the guidance of migrating neural crest-derived cells in the developing bowel and pancreas. *Dev Biol.* 258 364–384. 10.1016/s0012-1606(03)00136-2 12798294

[B21] Joseph N. He S. Quintana E. Kim Y. Nunez G. Morrison S. (2011). Enteric glia are multipotent in culture but primarily form glia in the adult rodent gut. *J. Clin. Invest.* 121 3398–3411. 10.1172/JCI58186 21865643 PMC3163971

[B22] Kuil L. Kakiailatu N. Windster J. Bindels E. Zink J. van der Zee G. (2023). Unbiased characterization of the larval zebrafish enteric nervous system at a single cell transcriptomic level. *iScience* 26:107070. 10.1016/j.isci.2023.107070 37426341 PMC10329177

[B23] Laranjeira C. Sandgren K. Kessaris N. Richardson W. Potocnik A. Vanden Berghe P. (2011). Glial cells in the mouse enteric nervous system can undergo neurogenesis in response to injury. *J. Clin. Invest.* 121 3412–3424. 10.1172/JCI58200 21865647 PMC3163972

[B24] Lasrado R. Boesmans W. Kleinjung J. Pin C. Bell D. Bhaw L. (2017). Lineage-dependent spatial and functional organization of the mammalian enteric nervous system. *Science* 356 722–726. 10.1126/science.aam7511 28522527

[B25] Lefevre M. Soret R. Pilon N. (2023). Harnessing the power of enteric glial cells’ plasticity and multipotency for advancing regenerative medicine. *Int. J. Mol. Sci.* 24:12475. 10.3390/ijms241512475 37569849 PMC10419543

[B26] Liu M. Kuan Y. Wang J. Hen R. Gershon M. (2009). 5-HT4 receptor-mediated neuroprotection and neurogenesis in the enteric nervous system of adult mice. *J. Neurosci.* 29 9683–9699. 10.1523/JNEUROSCI.1145-09.2009 19657021 PMC2749879

[B27] Majd H. Samuel R. Ramirez J. Kalantari A. Barber K. Ghazizadeh Z. (2022). hPSC-derived enteric ganglioids model human ENS development and function. *bioRxiv* [Preprint]. 10.1101/2022.01.04.474746

[B28] McCallum S. Obata Y. Fourli E. Boeing S. Peddie C. Xu Q. (2020). Enteric glia as a source of neural progenitors in adult zebrafish. *Elife* 9:e56086. 10.7554/eLife.56086 32851974 PMC7521928

[B29] McKeown S. Chow C. Young H. (2001). Development of the submucous plexus in the large intestine of the mouse. *Cell Tissue Res.* 303 301–305. 11291776 10.1007/s004410000303

[B30] Mueller J. Stavely R. Guyer R. Soos A. Bhave S. Han C. (2024). Agrin inhibition in enteric neural stem cells enhances their migration following colonic transplantation. *Stem Cells Transl. Med.* 13 490–504. 10.1093/stcltm/szae013 38387006 PMC11092276

[B31] Pilon N. (2021). Treatment and prevention of neurocristopathies. *Trends Mol. Med.* 27 451–468. 33627291 10.1016/j.molmed.2021.01.009

[B32] Progatzky F. Pachnis V. (2022). The role of enteric glia in intestinal immunity. *Curr. Opin. Immunol.* 77:102183. 10.1016/j.coi.2022.102183 PMC9586875 35533467

[B33] Progatzky F. Shapiro M. Chng S. Garcia-Cassani B. Classon C. Sevgi S. (2021). Regulation of intestinal immunity and tissue repair by enteric glia. *Nature* 599 125–130. 34671159 10.1038/s41586-021-04006-z PMC7612231

[B34] Rao M. Nelms B. Dong L. Salinas-Rios V. Rutlin M. Gershon M. (2015). Enteric glia express proteolipid protein 1 and are a transcriptionally unique population of glia in the mammalian nervous system. *Glia* 63 2040–2057. 10.1002/glia.22876 26119414 PMC4695324

[B35] Rao M. Rastelli D. Dong L. Chiu S. Setlik W. Gershon M. (2017). Enteric glia regulate gastrointestinal motility but are not required for maintenance of the epithelium in mice. *Gastroenterology* 153:1068–81.e7. 28711628 10.1053/j.gastro.2017.07.002 PMC5623141

[B36] Roberts R. Murphy J. Young H. Bornstein J. (2007). Development of colonic motility in the neonatal mouse-studies using spatiotemporal maps. *Am. J. Physiol. Gastrointest. Liver Physiol.* 292 G930–G938. 10.1152/ajpgi.00444.2006 17158255

[B37] Rosenberg H. Rao M. (2021). Enteric glia in homeostasis and disease: From fundamental biology to human pathology. *iScience* 24:102863. 10.1016/j.isci.2021.102863 34401661 PMC8348155

[B38] Schneider S. Wright C. Heuckeroth R. (2019). Unexpected roles for the second brain: Enteric nervous system as master regulator of bowel function. *Annu. Rev. Physiol.* 81 235–259. 10.1146/annurev-physiol-021317-121515 30379617

[B39] Seguella L. Gulbransen B. (2021). Enteric glial biology, intercellular signalling and roles in gastrointestinal disease. *Nat. Rev. Gastroenterol. Hepatol.* 18 571–587. 33731961 10.1038/s41575-021-00423-7 PMC8324524

[B40] Sharkey K. Mawe G. (2023). The enteric nervous system. *Physiol. Rev.* 103 1487–1564. 36521049 10.1152/physrev.00018.2022 PMC9970663

[B41] Singh S. Shariff A. Roy T. Das T. Rani N. (2015). Development of myenteric plexus in human foetuses: A quantitative study. *Anat. Cell Biol.* 48 124–129. 10.5115/acb.2015.48.2.124 26140223 PMC4488640

[B42] Soret R. Lassoued N. Bonnamour G. Bernas G. Barbe A. Pelletier M. (2021). Genetic background influences severity of colonic Aganglionosis and response to GDNF enemas in the holstein mouse model of Hirschsprung disease. *Int. J. Mol. Sci.* 22:13140. 10.3390/ijms222313140 34884944 PMC8658428

[B43] Soret R. Schneider S. Bernas G. Christophers B. Souchkova O. Charrier B. (2020). Glial cell derived neurotrophic factor induces enteric neurogenesis and improves colon structure and function in mouse models of Hirschsprung disease. *Gastroenterology* 159:1824–38.e17. 10.1053/j.gastro.2020.07.018 32687927

[B44] Sovrea A. Bosca A. (2013). Astrocytes reassessment - an evolving concept part one: Embryology, biology, morphology and reactivity. *J. Mol. Psychiatry* 1:18. 10.1186/2049-9256-1-18 26019866 PMC4445578

[B45] Srinivas S. Watanabe T. Lin C. William C. Tanabe Y. Jessell T. (2001). Cre reporter strains produced by targeted insertion of EYFP and ECFP into the ROSA26 locus. *BMC Dev. Biol.* 1:4. 10.1186/1471-213x-1-4 11299042 PMC31338

[B46] Tabata H. (2015). Diverse subtypes of astrocytes and their development during corticogenesis. *Front. Neurosci.* 9:114. 10.3389/fnins.2015.00114 25904839 PMC4387540

[B47] Uesaka T. Nagashimada M. Enomoto H. (2015). Neuronal differentiation in schwann cell lineage underlies postnatal neurogenesis in the enteric nervous system. *J. Neurosci.* 35 9879–9888. 10.1523/JNEUROSCI.1239-15.2015 26156989 PMC6605410

[B48] Uesaka T. Okamoto M. Nagashimada M. Tsuda Y. Kihara M. Kiyonari H. (2021). Enhanced enteric neurogenesis by Schwann cell precursors in mouse models of Hirschsprung disease. *Glia* 69 2575–2590. 10.1002/glia.24059 34272903

[B49] Windster J. Kuil L. Kakiailatu N. Antanaviciute A. Sacchetti A. MacKenzie K. (2023). Human enteric glia diversity in health and disease: New avenues for the treatment of Hirschsprung disease. *bioRxiv* [Preprint]. 10.1101/2023.09.26.559481 39725172

[B50] Woods C. Kapur R. Bischoff A. Lovell M. Arnold M. Pena A. (2021). Neurons populating the rectal extrinsic nerves in humans express neuronal and Schwann cell markers. *Neurogastroenterol. Motil.* 33:e14074. 10.1111/nmo.14074 33382200

[B51] Yoo B. Mazmanian S. (2017). The enteric network: Interactions between the immune and nervous systems of the gut. *Immunity* 46 910–926. 28636959 10.1016/j.immuni.2017.05.011 PMC5551410

[B52] Young H. Ciampoli D. (1998). Transient expression of neuronal nitric oxide synthase by neurons of the submucous plexus of the mouse small intestine. *Cell Tissue Res.* 291 395–401. 9477296 10.1007/s004410051009

