# Supplementary material for: Enteric glial cell diversification is influenced by spatiotemporal factors and source of neural progenitors in mice
Source: Front Neurosci. 2024 Aug 29;18:1392703. doi: 10.3389/fnins.2024.1392703 (PMC11390640; doi:10.3389/fnins.2024.1392703)
Supplement: Supplementary file 3 [file Data_Sheet_1.docx]

Supplementary Material

# Supplementary Figures

**Figure S1.** **Overview of experimental design and approaches for identifying topo-morphological subtypes of EGCs and quantifying structural changes in the ENS.**

**Figure S2. Morphological evolution of EGC subtypes between P1 and P20.**

**Figure S3. The identification of submucosal ganglia is facilitated by DAPI staining of neuronal nuclei.**

**Figure S4. Comparison of EGC subtype proportions in the submucosal plexus at early postnatal stages, using either SOX10 data alone or combined SOX10-S100β data.**

**Figure S5. The *Dhh-Cre*;*Rosa26^[FloxedSTOP]YFP^* system specifically labels extrinsic SCPs and none of the intrinsic ENS progenitors directly derived from neural crest cells.**

**Figure S6.** C**ontribution of SCPs to topo-morphological EGC subtypes, detailed per sample.**

**Figure S7. MPZ is not detected in SCP-derived EGCs from the distal colon of P15 mice.**

**
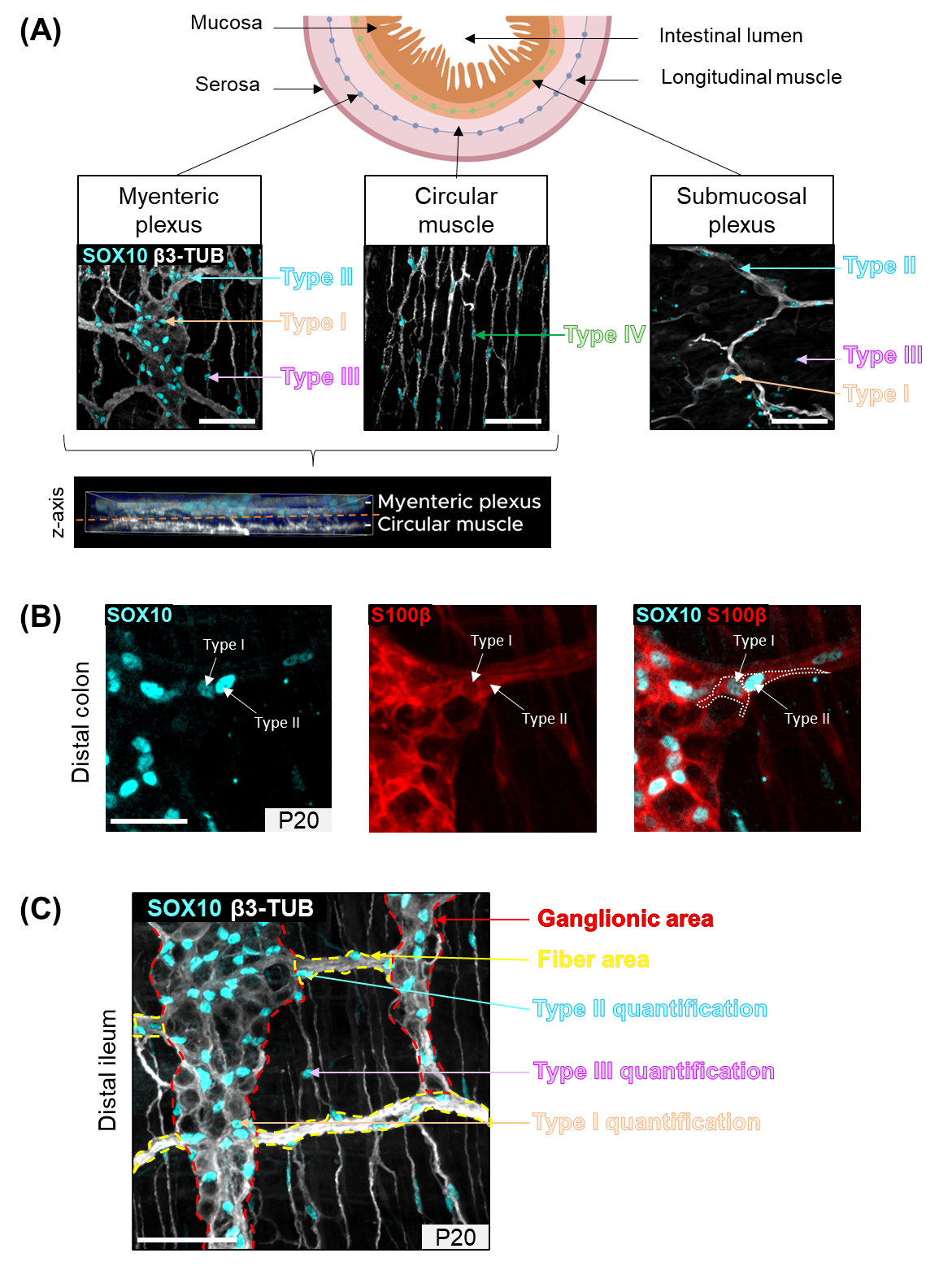
**

**Figure S1.** **Overview of experimental design and approaches for identifying topo-morphological subtypes of EGCs and quantifying structural changes in the ENS.**

**(A)** Schematic of the classification system used to categorize EGC subtypes based on topological criteria. Type I are located in ganglia, Type II at the border of and within interganglionic fibers, Type III in the same plane of the plexuses but outside ganglia and fibers, and Type IV deep in the muscle layer. **(B)** Immunofluorescence staining of P20 distal colon showing how to discriminate between Type II EGCs at fiber edges from Type I EGCs, based on their respective morphology. Type I EGCs exhibit multiple processes extending in different directions whereas Type II exhibit fibrous processes that run in parallel to interganglionic fibers. Tissue was immunolabeled with antibodies against the nuclear EGC marker SOX10 and the cytoplasmic EGC marker S100β, allowing to discern gross EGC morphology. **(C)** Immunofluorescence staining of P20 distal colon with SOX10 and βIII-Tubulin antibodies, showing the methodology for quantification of EGC subtypes and morphometric parameters. Scale bar, 70 µm (A, C) or 35 µm (B)


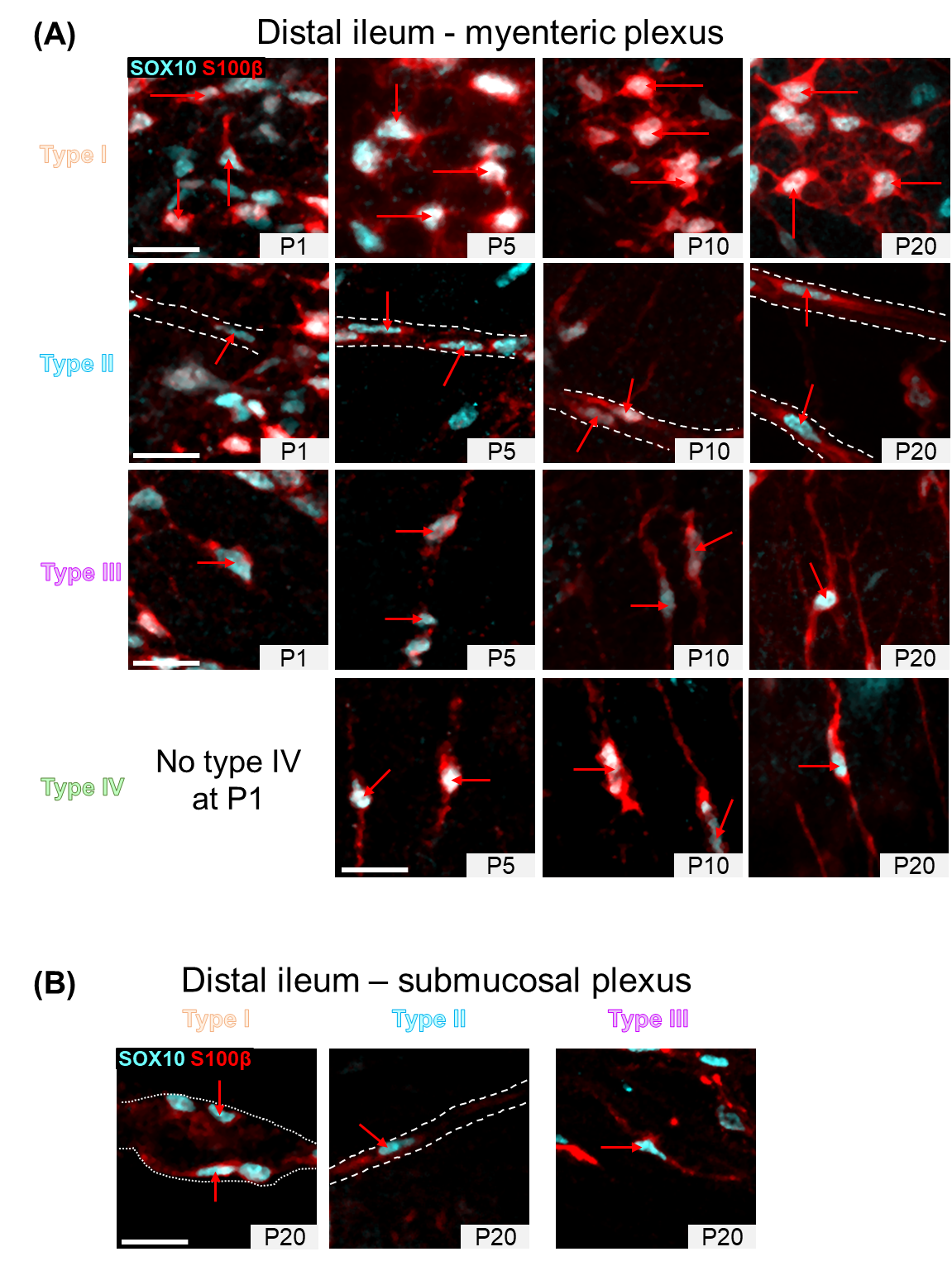


**Figure S2. Morphological evolution of EGC subtypes between P1 and P20.**

**(A-B)** Representative images of SOX10+ S100β+ EGC subtypes showing that each subtype gradually acquires its main gross morphological attributes (multipolar Type I, elongated Type II, multipolar Type III, bipolar Type IV) before P20 in the myenteric plexus of the distal ileum (A), but not yet in the submucosal plexus of the same bowel segment (B). Scale bar, 20 µm

**
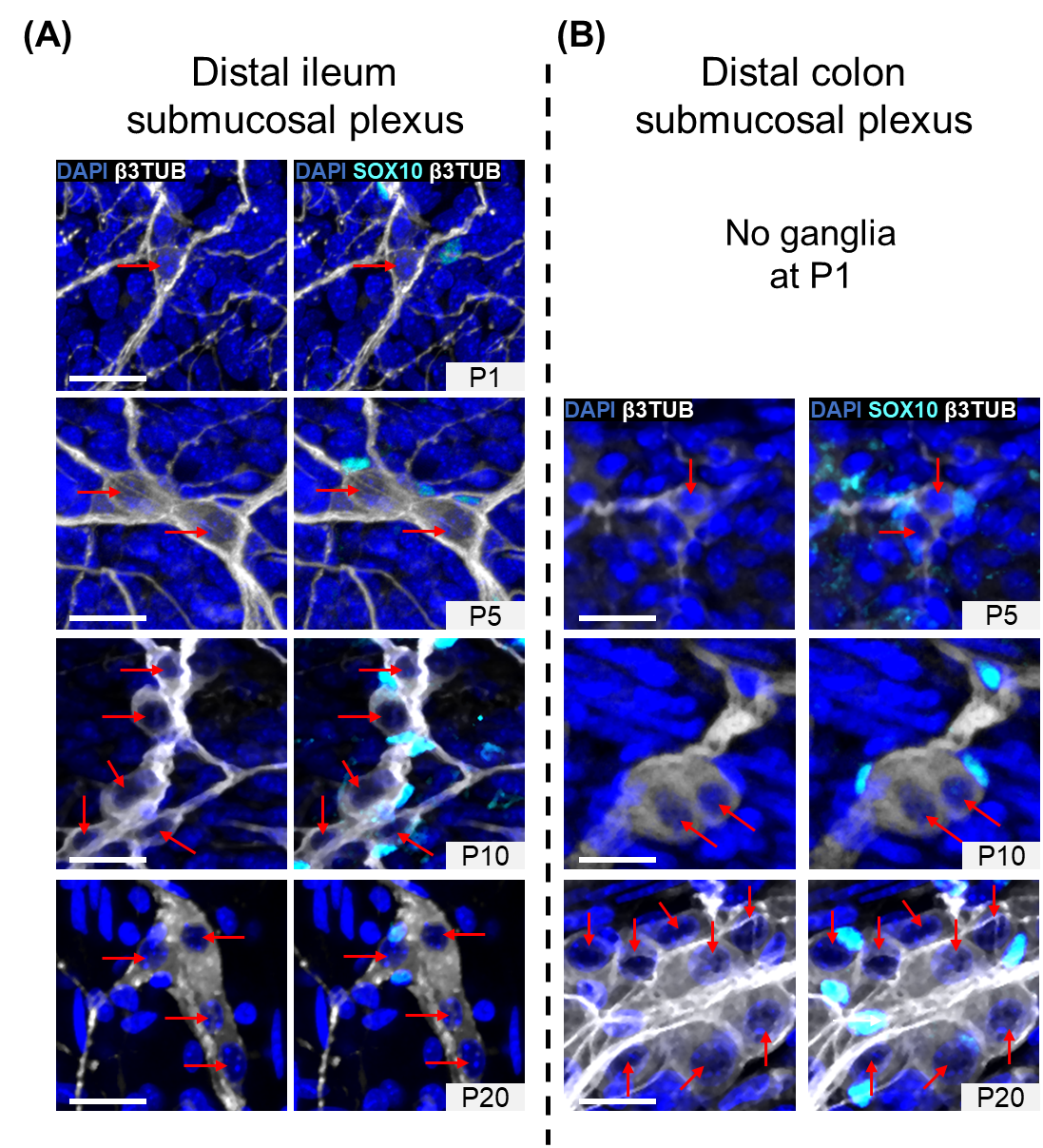
Figure S3. The identification of submucosal ganglia is facilitated by DAPI staining of neuronal nuclei.**

**(A-B)** Representative images of β3TUB+ neuronal networks showing that DAPI staining allows to distinguish the round nucleus of neurons (arrows) in submucosal ganglia of variable sizes (≥1 neuron) between P1 and P20, in both the distal ileum (A) and distal colon (B). Scale bar, 20 µm.

**
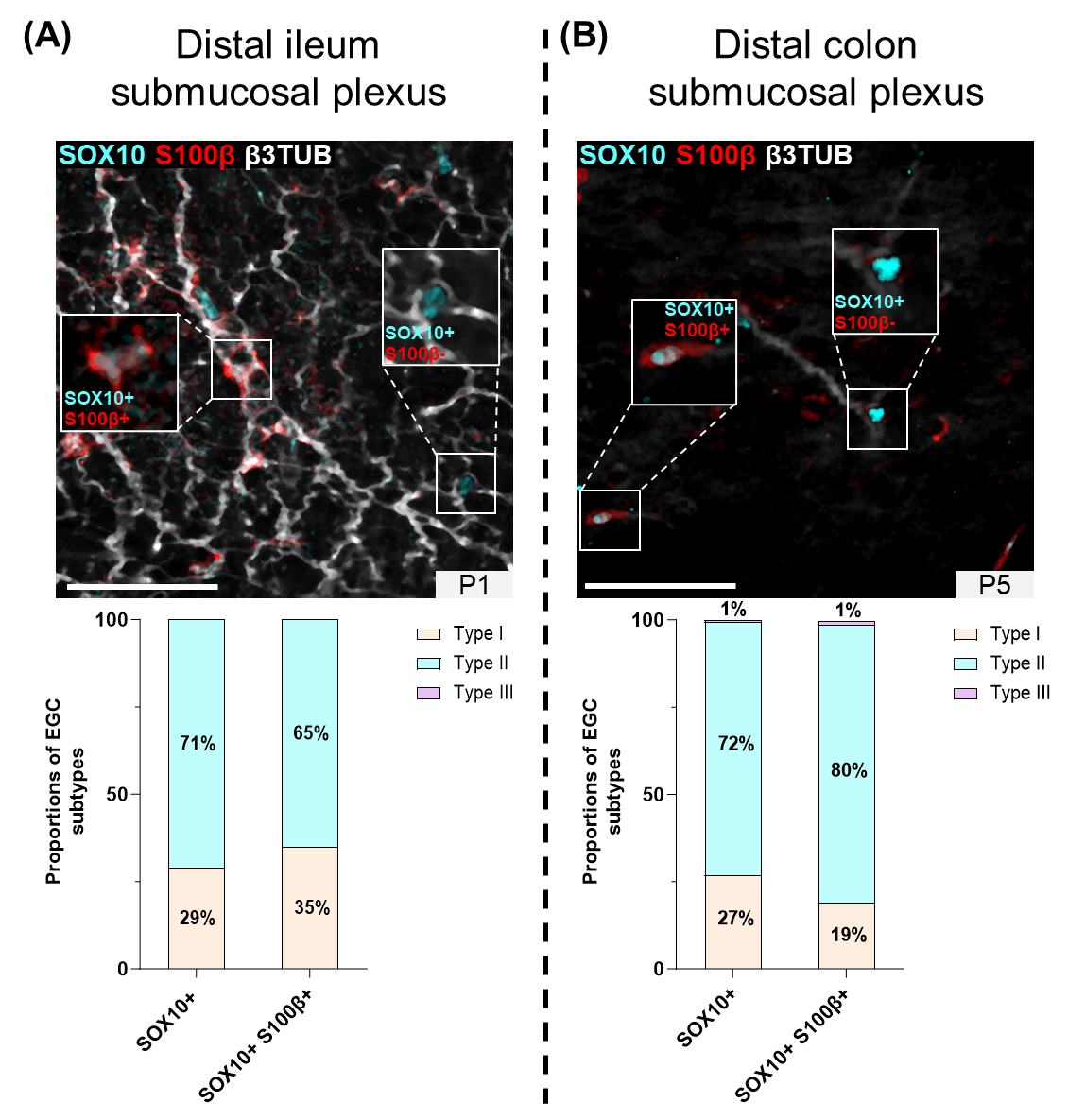
Figure S4. Comparison of EGC subtype proportions in the submucosal plexus at early postnatal stages, using either SOX10 data alone or combined SOX10-S100β data.**

**(A-B)** Representative images of EGCs that stain positive for either SOX10 alone or for both SOX10 and S100β (insets in upper panels), and accompanying quantifications (lower panels) of EGC Type I, Type II and Type III in the submucosal plexus of the distal ileum at P1 (A) and distal colon at P5 (B). Scale bar, 70 µm.

**
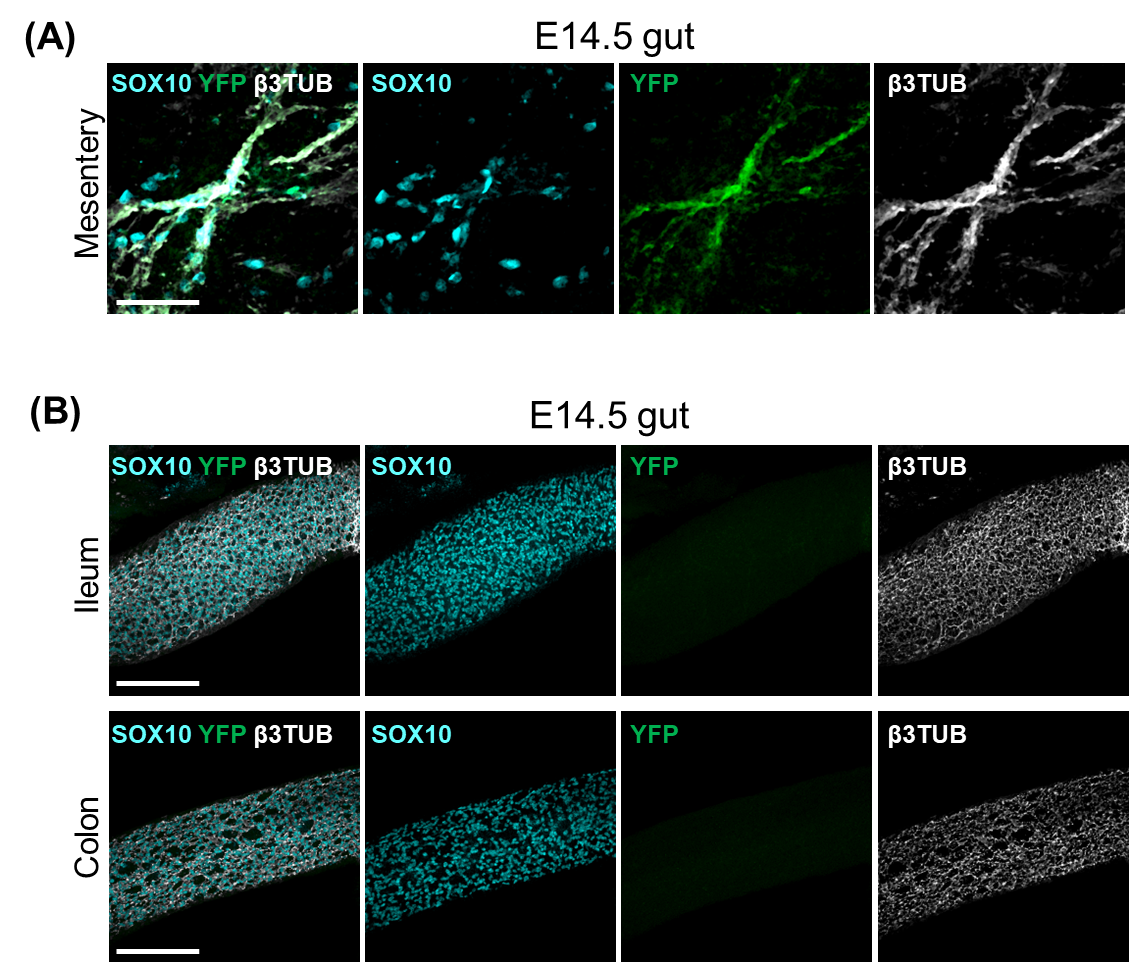
**

**Figure S5. The *Dhh-Cre*;*Rosa26^[FloxedSTOP]YFP^* system specifically labels extrinsic SCPs and none of the intrinsic ENS progenitors directly derived from neural crest cells.**

**(A-B)** Representative images of e14.5 embryonic guts showing that YFP is detected in a subset of SOX10+ cells along extrinsic β3TUB+ mesentery-associated nerves (A) but not in any of the intrinsic tissue-resident SOX10+ ENS progenitors in the distal ileum and distal colon (B). Scale bar, 70 µm (A) or 200 µm (B).


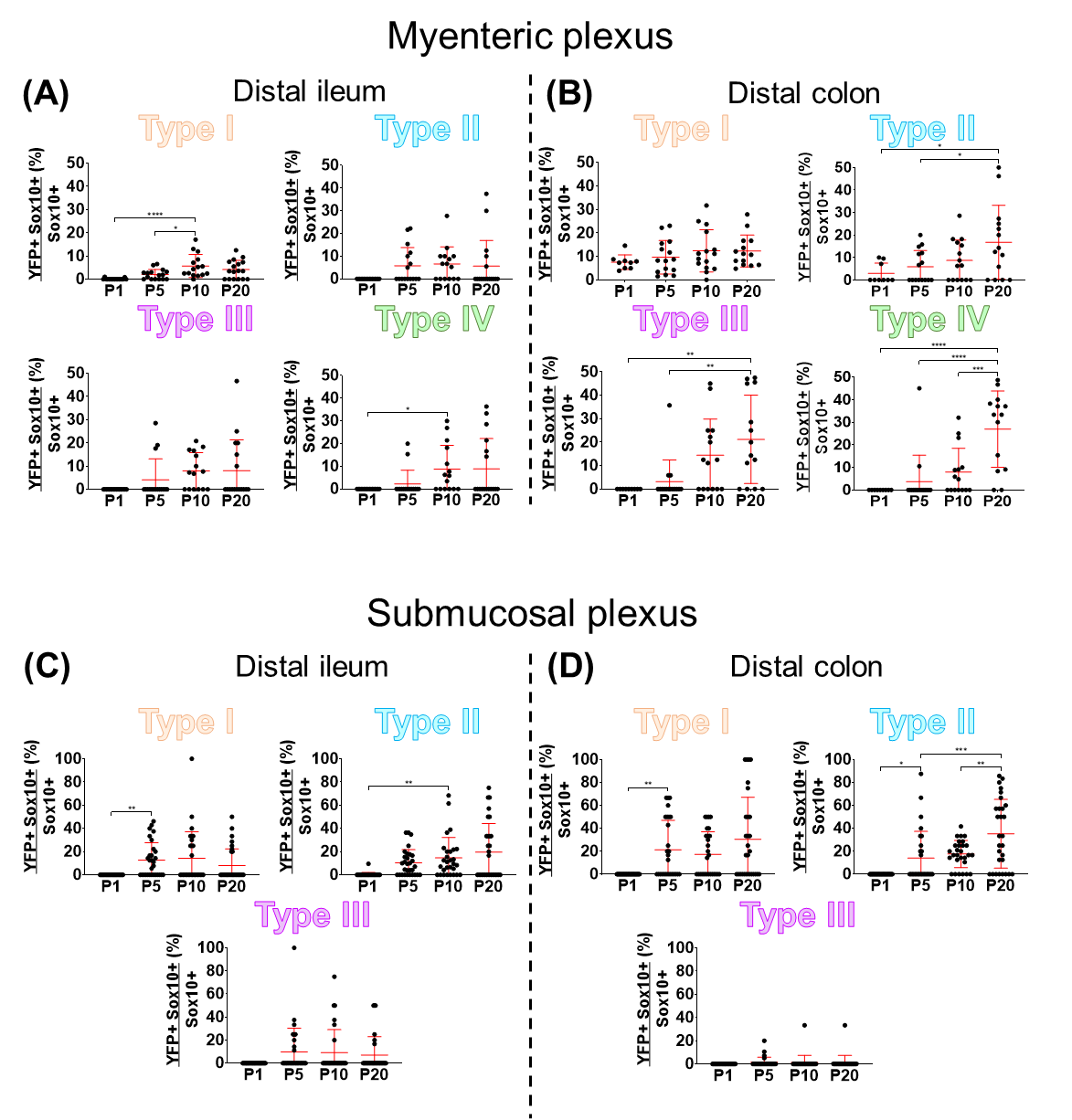
**Figure S6.** C**ontribution of SCPs to topo-morphological EGC subtypes, detailed per sample.**

(**A-D)** Quantitative analysis of YFP+ SOX10+ EGCs proportion among total SOX10+ EGC Type I, II, III or IV, between P1 and P20, in the myenteric plexus of the distal ileum **(A)** and of the distal colon **(B)**, as well as in the submucosal plexus of the distal ileum **(C)** and of the distal colon **(D).** Each dot represents the percentage of YFP+ SOX10+ EGC Type I, II, III or IV in a single 60X field of view (N=3 mice per time point, n=3-5 fields of view per tissue for the myenteric plexus/muscular layer; n= 6-10 fields of view per tissue for the submucosal layer). **P*≤0.05, ***P*≤0.01, ****P*≤0.001, *****P*≤0.0001; One-Way ANOVA and Tukey’s multiple comparison test.

**
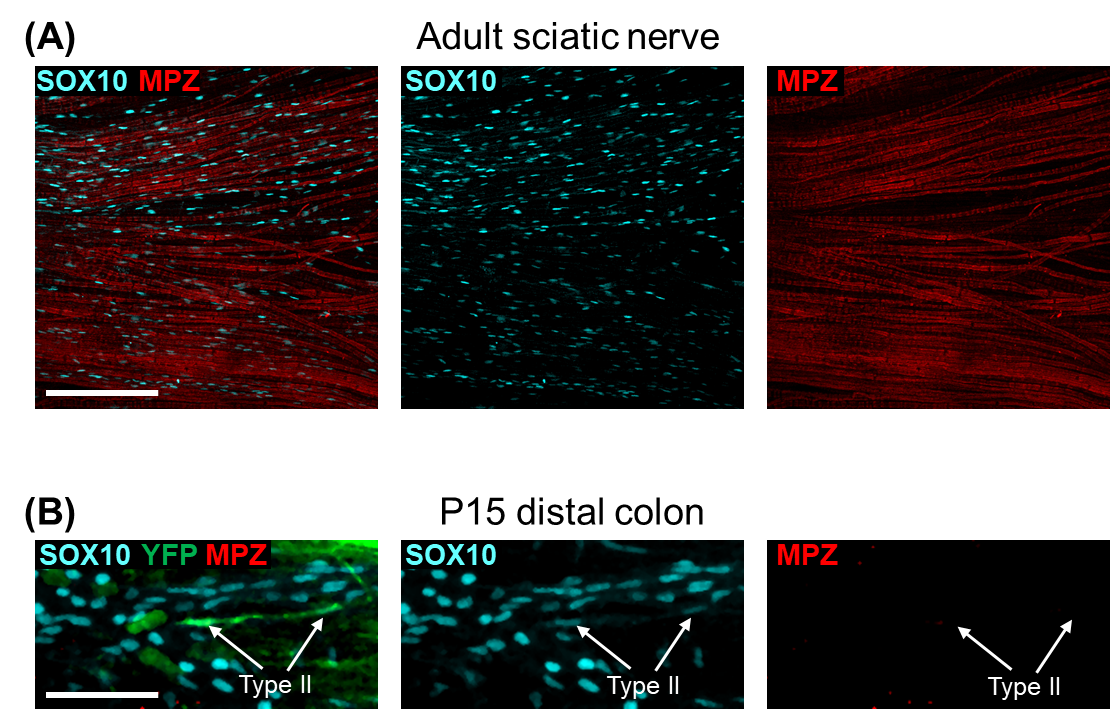
**

**Figure S7. MPZ is not detected in SCP-derived EGCs from the distal colon of P15 mice.**

**(A-B)** Immunofluorescence analysis of the adult sciatic nerve from WT FVB mice **(A)** and myenteric plexus from the distal colon of P15 *Dhh*-Cre;*Rosa26^[FloxedSTOP]YFP^* mice **(B)**. Tissues were immunolabeled with antibodies against SOX10 (cyan), MPZ (red) and/or GFP/YFP (green). Displayed images are z-stack projections representative of observations made from N=3 mice. Scale bar, 200μm (A) or 50µm (B).

# Supplementary Tables

**Table S1. Oligonucleotide primers used for genotyping.**

| **GENE** | **SENSE PRIMER** | **ANTISENSE PRIMER** |
| --- | --- | --- |
| ***Rosa26-YFP*** | 5’CCCAAAGTCGCTCTGAGTTGTTATC’ | YFP: 5’TGCGCCCTACAGATCCCTTAATTAA3’  WT: 5’CCAGATGACTACCTATCCTCCCA3’ |
| ***Dhh-Cre*** | 5’GATGAGGTTCGCAAGAACCTGATG3’ | 5’AACAGCATTGCTGTCACTTGGTCG3’ |

**Table S2. Primary and secondary antibodies used for immunofluorescence.**

| **ANTIBODY** | **SOURCE** | **HOST SPECIES** | **DILUTION** |
| --- | --- | --- | --- |
| **SOX10** | Pilon lab (made by Medimabs) | Rat | 1:500 |
| **SOX10** | R&D Systems, #AF2864 | Goat | 1:500 |
| **S100β** | Novus Biologicals, #NBP1-41373SS | Rabbit | 1:500 |
| **βIII-Tubulin** | Abcam, #ab78078 | Mouse | 1:500 |
| **Green Fluorescent Protein (GFP)** | Abcam, #ab290 | Rabbit | 1:500 |
| **Green Fluorescent Protein (GFP), N-terminal** | Sigma-Aldrich, #G6795 | Mouse | 1:500 |
| **Myelin Protein Zero (MPZ)** | Abcam, #ab31851 | Rabbit | 1:500 |
| **AlexaFluor 488 Anti-rat** | Jackson ImmunoResearch Laboratories Inc, 712-545-151 | Donkey | 1:500 |
| **AlexaFluor 594 Anti-rabbit** | Jackson ImmunoResearch Laboratories Inc, 711-585-152 | Donkey | 1:500 |
| **AlexaFluor 647 Anti-mouse** | Jackson ImmunoResearch Laboratories Inc, 715-605-150 | Donkey | 1:500 |
| **AlexaFluor 488 Anti-goat** | Jackson ImmunoResearch Laboratories Inc, 805-545-180 | Bovine | 1:500 |

# Supplementary Movies

**Movie S1. Animated 3D projection of z-stack micrographs of the myenteric plexus and circular muscle immunolabeled with SOX10 and βIII-Tubulin antibodies.**

**Movie S2. Animated 2D view of z-stack micrographs of the myenteric plexus and circular muscle immunolabeled with SOX10 and βIII-Tubulin antibodies.**
